# Thumb Reconstruction with Arthrodesis to the Second Metacarpal following Sarcoma Excision

**DOI:** 10.1155/2016/8061036

**Published:** 2016-08-29

**Authors:** Christopher Hein, Barry Watkins, Lee M. Zuckerman

**Affiliations:** Department of Orthopaedic Surgery, Loma Linda University Medical Center, 11406 Loma Linda Drive, Suite 218, Loma Linda, CA 92354, USA

## Abstract

Primary sarcomas of the thumb metacarpal are rare malignant lesions. Surgical treatment involves amputation versus tumor resection with thumb reconstruction. If complete tumor resection is possible, thumb preservation may be considered, as the thumb is vital to hand function. Following tumor resection, previous reports have described graft reconstruction with fusion to the trapezium or scaphoid. We present two cases of sarcoma necessitating resection of the thumb metacarpal that were reconstructed with an arthrodesis of the proximal phalanx to the second metacarpal shaft. Arthrodesis to the second metacarpal allows robust bony contact for fusion as well as improved resting position of the thumb. At 2- and 4-year follow-up, both patients have a stable, pain-free thumb without evidence of local recurrence.

## 1. Introduction

Primary bony tumors of the thumb metacarpal are a rare and challenging problem [1]. Chondrosarcoma is the most common malignant tumor of the hand but typically arises in the metacarpal or proximal phalanx of the index or small finger [2]. Synovial sarcoma is even less common in the fingers and usually presents in the wrist or hand. Historically, ray resection represented the foundation of treatment for malignant metacarpal lesions. However, ray resection of the thumb results in a greater functional deficit as compared with the resection of the other digits [3]. Thumb reconstruction with allograft or autograft has been shown to be a reasonable treatment option if acceptable margins are feasible and vital neurologic structures are spared [1, 3–8]. Previous descriptions of reconstructive efforts include iliac crest, fibula, and ulna autograft arthrodesis to the carpus. We report two cases of sarcoma that required resection of the entire thumb metacarpal, including one chondrosarcoma and one synovial cell sarcoma. Both were treated by resection and autograft arthrodesis of the proximal phalanx of the thumb to the shaft of the second metacarpal. To our knowledge, this technique has not been previously described after tumor resection.

## 2. Case Reports

### 2.1. Case  1

**Figure 1:**
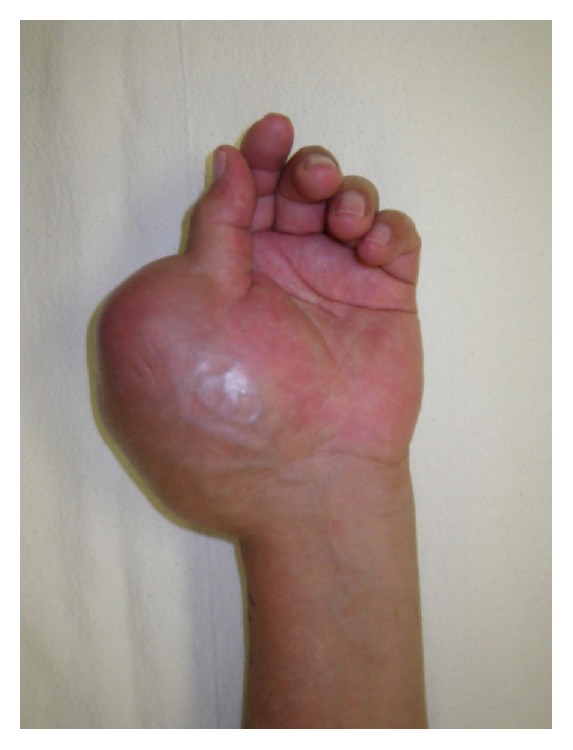
Preoperative photograph of the left hand demonstrating a large thumb mass.

A 60-year-old male was referred to the orthopaedic clinic for an enlarging left hand mass (Figure 1). The hard, originally painless mass was located on the dorsum of the left thumb metacarpal and had been present for 6 years. The mass began enlarging and became painful. Its size began to affect the patient's thumb function. Physical examination showed a large, firm, minimally tender mass on the dorsoradial aspect of the hand over the thumb metacarpal which measured approximately 9 cm. Interphalangeal (IP) joint motion was decreased from 0 to 30 degrees, and thumb abduction and opposition were minimal. He had no lymphadenopathy, no systemic symptoms, and normal laboratory values.

**Figure 2:**
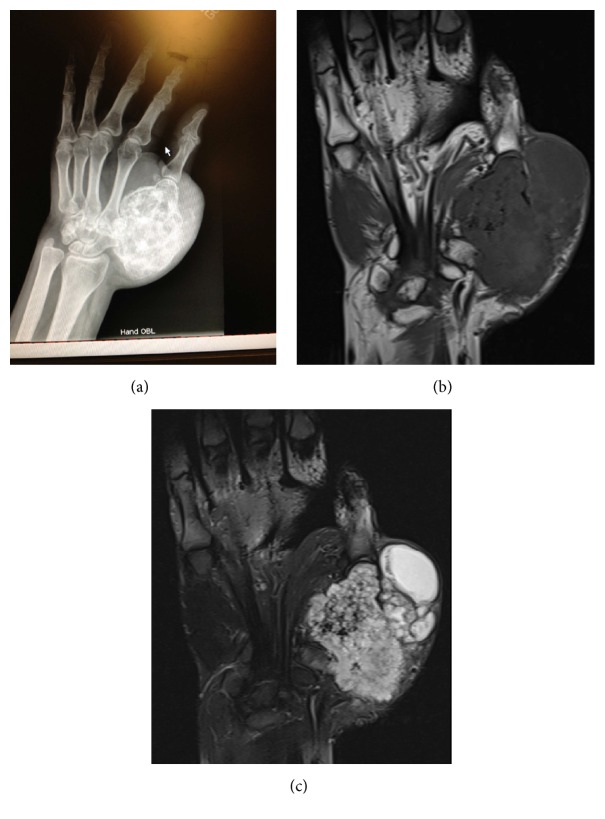
Preoperative imaging. (a) X-rays demonstrating a large, calcified tumor arising from the first metacarpal. (b) Coronal T1-weighted MRI of the left hand. (c) Coronal T2-weighted MRI of the left hand.

Plain radiographs (Figure 2(a)) revealed a large, partially calcified mass arising from the thumb metacarpal. T1-weighted magnetic resonance imaging (MRI) (Figure 2(b)) showed a heterogeneous, expansile mass measuring 6 × 9 cm arising from the thumb metacarpal with complete involvement of the trapezium. T2-weighted imaging (Figure 2(c)) revealed a mass with increased signal and cystic, loculated regions without tendon sheath enhancement. A whole body bone scan and computed tomography (CT) scan of chest, abdomen, and pelvis revealed no other lesions. A core biopsy was consistent with a Grade 2 chondrosarcoma.

Surgical treatment involved en bloc resection of the affected metacarpal with a portion of the proximal phalanx of the thumb and the entire trapezium in order to obtain negative margins. The extensor carpi radialis longus (ECRL), extensor pollicis longus (EPL), and flexor carpi radialis (FCR) were resected with the tumor. The flexor pollicis longus was uninvolved and therefore preserved. Tricortical iliac crest autograft was utilized for reconstruction of the proximal thumb and secured distally to the first proximal phalanx and proximally to the second metacarpal with two 2.7 mm locking plates and a lag screw in a position of opposition to the index finger. The EPL was reconstructed with free FCR autograft which was harvested proximally, away from the zone of tumor involvement. To prevent bowstringing of the extensor tendon, the ECRL was then harvested proximal to the area of tumor involvement and was utilized to reconstruct the extensor retinaculum.

**Figure 3:**
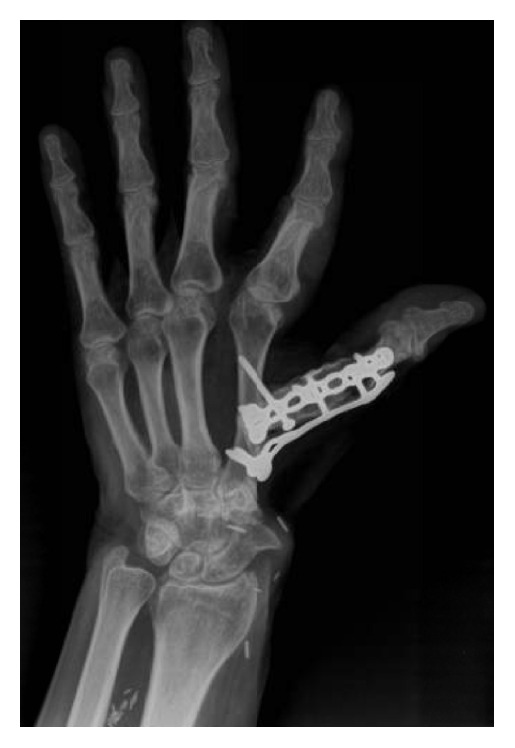
Postoperative X-ray of the left hand demonstrating fusion of the proximal phalanx of the thumb to the second metacarpal.

**Figure 4:**
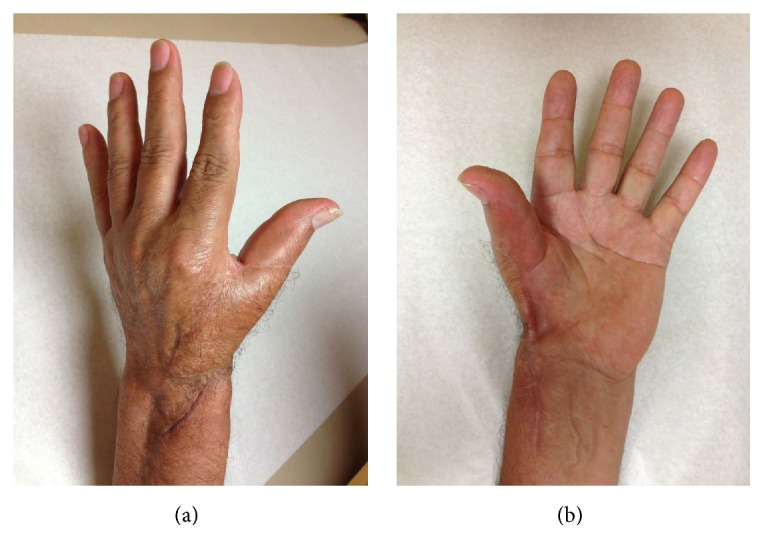
Postoperative clinical photographs. (a) Dorsal view. (b) Palmar view.

Postoperative pathology confirmed clear surgical margins. The postoperative course was complicated by necrosis of the skin and a wound dehiscence which resulted in exposed hardware. An irrigation and debridement were performed resulting in a wound measuring 9 × 6 cm. Coverage of the wound was obtained with a free fasciocutaneous flap from the dorsal forearm based on the posterior interosseous artery and anastomosed to the radial artery. Most recent follow-up at 4 years postoperatively revealed a stable, pain-free thumb. His IP joint had evidence of degenerative changes and his motion was limited from 0 to 5 degrees compared to 0 to 30 degrees preoperatively. There were no radiographic or clinical signs of recurrent disease. There was radiologic (Figure 3) and clinical evidence of a successful arthrodesis (Figures 4(a) and 4(b)). A Disabilities of the Arm, Shoulder and Hand (DASH) score was 13.3 and the Musculoskeletal Tumor Society (MSTS) score was 25.

### 2.2. Case  2

**Figure 5:**
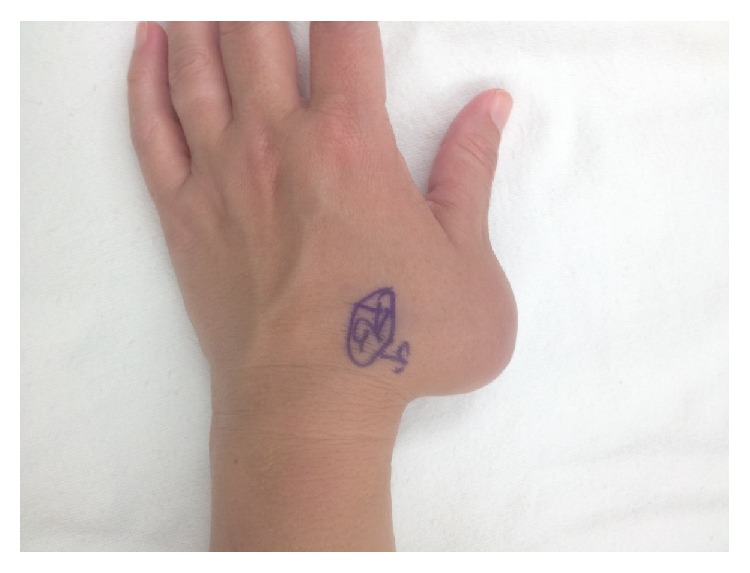
Preoperative photograph of the left hand demonstrating a large thumb mass.

A 39-year-old female was referred to the orthopaedic clinic for a left hand mass that had been enlarging over the past 9 months (Figure 5). Physical examination revealed a large, soft mass overlying the palmar and radial aspect of the thumb metacarpal with mild tenderness and intact thumb motion. Sensation and vascular status were normal. She had no fever or systemic symptoms and normal laboratory values.

**Figure 6:**
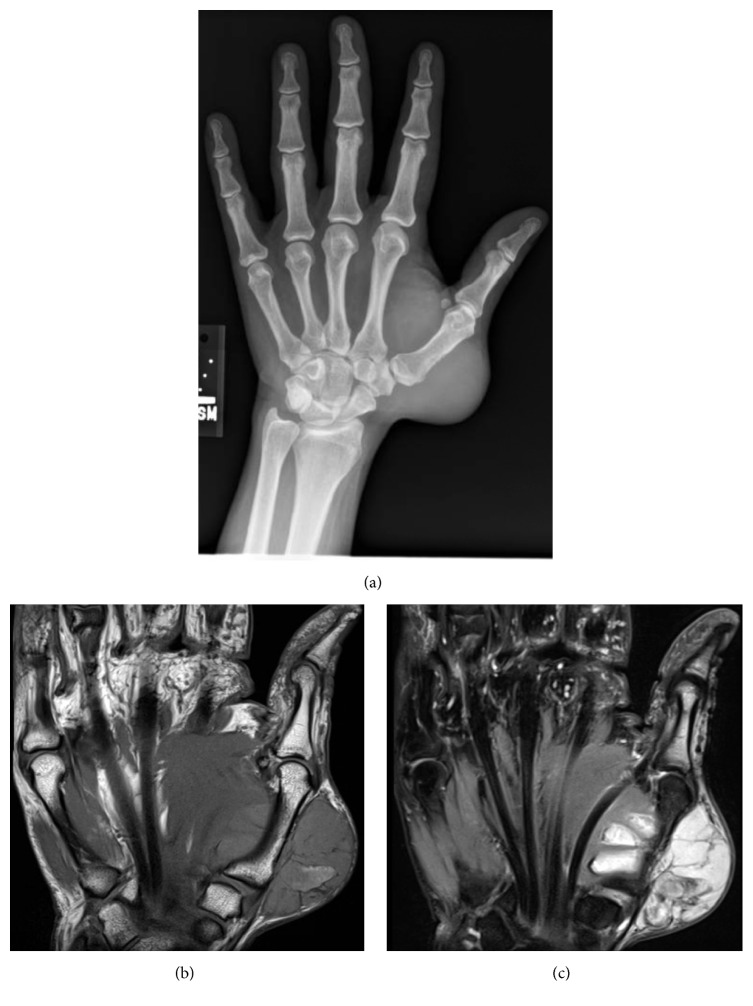
Preoperative imaging. (a) X-rays demonstrating a soft tissue mass with some underlying bony erosion of the first metacarpal. (b) Coronal T1-weighted MRI of the left hand demonstrating envelopment of the first metacarpal. (c) Coronal T2-weighted MRI of the left hand demonstrating involvement of the first metacarpal.

Plain radiographs revealed a large, noncalcified soft tissue mass adjacent to the thumb metacarpal with some metacarpal erosion (Figure 6(a)). T1-weighted MRI sequences showed a large heterogeneous mass encompassing the thumb metacarpal with focal signal change in the metacarpal (Figure 6(b)). On T2-weighted imaging, the mass had increased signal and was multilobulated (Figure 6(c)). There was enhancement of the flexor pollicis longus and extensor tendon sheaths.

A CT of the chest was normal and there was no lymphadenopathy. An open biopsy of the tumor revealed a highly cellular proliferation of spindle cells in sheets consistent with a high grade synovial cell sarcoma.

**Figure 7:**
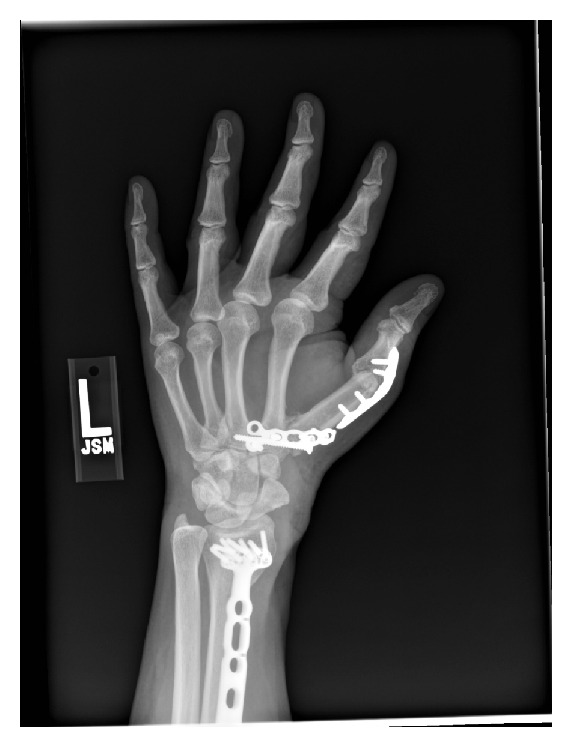
Postoperative X-ray of the left hand.

Surgical resection with a staged reconstruction was then performed. A staged reconstruction was chosen in this case to confirm negative margins prior to undergoing reconstruction. A vascularized autograft was chosen to optimize the chance of arthrodesis in the setting of planned adjuvant radiation therapy. The entire thumb metacarpal, trapezium, flexor pollicis longus, EPL, and first dorsal compartment tendons were resected and the wound was closed with provisional K-wire fixation of the thumb. Pathologic examination revealed negative margins. One week later, the patient returned to the operating room for reconstruction. The metacarpal reconstruction was performed with a 5.5 cm distal radial osteofasciocutaneous flap vascularized by the radial artery. The graft was fixed proximally to the index metacarpal with a 2.4 mm locking plate and lag screw and distally to the proximal phalanx with a separate 2.4 mm locking plate. The graft was positioned to provide for index finger to thumb opposition with the thumb IP joint in flexion. Full flexion of the index finger to the palm was obtained when the thumb IP joint was extended. Approximately one-half of the distal radial shaft and metaphysis were utilized, and the defect was prophylactically stabilized with a contoured volar locking plate (Figure 7). An extensor indicis proprius to EPL tendon transfer was then performed to restore extension of the thumb.

**Figure 8:**
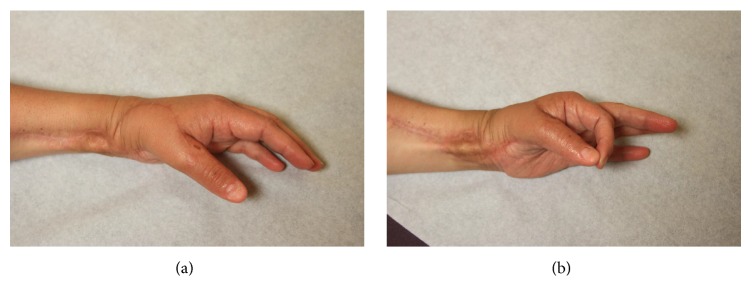
Postoperative clinical photographs. (a) Dorsal view of the thumb in a resting position. (b) Demonstration of the patient performing a functional pinch to the index finger.

The patient received adjuvant radiation to the area with a total of 50 grays of radiation. At 2 years, thumb IP range of motion was 0–70 degrees compared to 0–80 degrees preoperatively. She had functional pinch strength and no evidence of local recurrence (Figures 8(a) and 8(b)). DASH and MSTS scores were 17.5 and 24, respectively. Clinical and radiographic arthrodesis has been achieved.

## 3. Discussion

Chondrosarcoma involvement of the hands and feet comprise 1–3.2% of all chondrosarcomas, and most of these are phalangeal tumors [3, 9, 10]. Of 23 chondrosarcomas of the hand reported in the Scottish Bone Tumour Registry from 1954 to 1999, only one case involved the thumb metacarpal [10].

Synovial cell sarcoma is an uncommon soft tissue tumor that usually presents in the lower extremities. Deshmukh et al. reported only 5 synovial cell sarcomas occurring in the hand out of a total of 135 patients [11]. Successful treatment of these types of tumors with restoration of function is more complex then treating lesions of the other digits, as the thumb is integral to hand function [3]. Amputation of the thumb, while highly successful in preventing local recurrence, results in a significant functional deficit that is greater than amputation of the other digits [12]. Therefore, a concerted effort to maintain function while still performing a resection with negative surgical margins is the goal when treating malignant tumors of the thumb metacarpal.

Iliac crest, fibular allograft, free fibular autograft, and distal ulna have all been described for reconstruction after tumor removal at the first metacarpal [1, 5, 7, 8]. In these reports, the graft was secured to the trapezium or scaphoid proximally. In our case series, the tumors were large and necessitated resection of the trapezium in order to obtain negative margins. The options for proximal arthrodesis were therefore limited to the scaphoid or index metacarpal. Autograft was used in both cases. As the treatment plan for the patient with the chondrosarcoma did not involve adjuvant radiation, iliac crest was chosen. A vascularized autograft was chosen when adjuvant radiation was used in order to increase the rate of fusion. Both options resulted in a successful arthrodesis.

Although described previously with good results, osteosynthesis to the scaphoid presents multiple potential problems [6]. First, the bony surface area for arthrodesis between graft and scaphoid is small, decreasing the chance of a successful fusion. Second, the distance from the proximal base of the graft to the tip of the thumb is increased with fixation to the scaphoid necessitating a larger graft. This creates a longer lever arm with increased torque at the fusion sites, which may result in a nonunion or failure of the hardware. In addition to this, resection of the thenar musculature and fusion of the thumb results in loss of the carpometacarpal motion. A fusion to the scaphoid fixes the base of the thumb in extension, which makes palmar adduction or abduction for grip more difficult.

The natural resting position of the thumb is in opposition to the distal phalanx of the index finger with the metacarpophalangeal (MCP) and IP joints flexed 10–15 degrees [13]. With grasp and key pinch activities, axial rotation at the trapeziometacarpal joint places the thumb out of the plane of the hand and in a position of function. This motion is lost following reconstruction with arthrodesis of the MCP and trapeziometacarpal joints. Therefore, the optimal position of fusion is one where the thumb can be placed in a position of maximum function, which is in slight palmar abduction and external rotation. In this position, the thumb is able to participate in power grip and pinch with IP flexion. Our reconstructive technique utilized a fusion site to the index metacarpal shaft. Due to the size and shape of the metacarpal, this position was able to be achieved without difficulty. Other benefits include the increased surface area for the arthrodesis to occur. There is also an increased area for placement of hardware to the metacarpal than to the carpus, providing for a higher likelihood of a successful fusion and a more stable base to allow earlier participation in rehabilitation. The position of the thumb at rest is also able to be placed further outside the plane of the hand, which more closely resembles the natural resting position of the thumb. This enhances the cosmesis of the reconstruction. Lastly, for functional activities, including key pinch and grasp, IP flexion in a plane perpendicular to the hand most closely resembles the anatomic position of function during key pinch and grasping activities.

In conclusion, we described two cases of sarcoma, necessitating resection of the thumb metacarpal that were reconstructed with an arthrodesis of the proximal phalanx of the thumb to the index metacarpal. Fusion was achieved in both cases and both patients had a pain-free, functional, and cosmetically acceptable thumb with no local recurrence. Arthrodesis of the thumb to the second metacarpal is a viable treatment option after tumor resection.
